# Telepathology and continuous education: important tools for pathologists of developing countries

**DOI:** 10.1186/1746-1596-3-S1-S24

**Published:** 2008-07-15

**Authors:** Hugo Góngora Jará, Héctor A Barcelo

**Affiliations:** 1 Department of Histology and Pathology. School of Medicine. Instituto Universitario de Ciencias de la Salud Fundación H.A. Barceló. Department of Pathology. Hospital Regional Dr. E. Vera Barros. 5300-La Rioja, Argentina; 2 Departament of Pathology. School of Medicine. Rector of the University. Instituto Universitario de Ciencias de la Salud Fundación H.A. Barceló. La Rioja, Santo Tomé and Buenos Aires, Argentina

## Abstract

Education shows that active participation allows the best development of skills to acquire, and the results are better when the information is well documented. Now, with digital images and the Internet, in the case of the Static Telepathology (ST), it is easy to share macroscopic and microscopic photographs. The progress of the technologies enabled a form of Dynamic Telepathology (DT) named "virtual slides", with navigation tools, and can be moved around changing powers as desired, making any personal computer into a digital microscope. The use of these tools in continuous education leads to optimal development of knowledge. We reported the experience of a Latin-American Pathologist from La Rioja, a small Province of Argentina, and we also mentioned the electronic publications in Virtual Hispano-American Congresses of Pathology (VHACP) since 1997(18 reports in the case of ST) and in two Virtual Slide Congress (VSC). In the 1st (2005) and 2nd (2007) Internet VSCs two of our cases were digitized in Spain (case 1 and 3 respectively). In these Virtual Slides, the microscopic images can be moved remotely from any computer connected to the Internet, we should recognize that it will become a valuable continuing Medical Education tool in microscopy, probably related to the phrase "a picture is worth more than a thousand words", then we might add; "what about thousands of images?" Similarly, the autoevaluation test is very important. ST and DT, in support of Virtual Congresses allows learning, teaching and sharing of diseases in scientific presentations and the exchange of views in the forums, these are the optimum material for distance education. In addition we received CDs or DVDs and certificates as authors, recognized by European Institutions. The active participation and the autoevaluation test are the best tools for continuous medical education in telepathology, not only for pathologists in developing countries but for the entire world.

## Introduction

Telepathology is the transmission of digital images of Pathology by telecommunications systems at distance for the purpose of consultation, diagnosis, forum cases, virtual conferences, research or teaching. Education shows that active participation allows the best development of acquired skills and results are better when the information is well documented. Now, with the digital images and the Internet, in the case of the ST, it is easy to share macroscopic and microscopic photographs [1], and we will refer more specifically in use at the VHACP. The progress of technologies enabled "virtual slides" with navigation tools, and can be moved around, changing powers as desired, making any personal computer into a digital microscope, a form of DT and in this case we mention the virtual microscope or virtual slide with our participation in the 1st and 2 VSCs, which uses previously digitized images in all increases and are placed on a server. These plates are generated by virtual microscopy systems that are capable of fully digitizing the histological and cytological preparations or WSI (whole slide imaging). There are several systems available on the market. Physically Virtual Slide is a directory with a set of image files, often JPEG 2000, and a file of coordinates, which contains information of how to rebuild the slide [1]. The Virtual Congresses are scientific meetings on the Internet, in which scientific work is published in the form of Web pages. The International Association of Biomedical Sciences on the Internet INABIS organized the first global Virtual Congress in 1994. The first VHACP was performed in 1997, organized by the University of Las Palmas de Gran Canaria; we are on the eve of the tenth. Also two VSCs were conducted simultaneously in 2005 and 2007 [2]. These congresses are an excellent virtual library and the use of these tools in the continuing Medical Distance Education, lead to optimal development of knowledge [3].

## Materials and methods

To expose the experience of the Latino-American Pathologist from La Rioja, a small Province of Argentina, in 9 VHACP since 1997 and in two VSC (2005 and 2007) with Spain Server. For the first VHACP we needed an engineer publisher of Web pages and the first digital images we obtained were scanned from paper photos. These pictures had at least 1 MB each and when sent by e-mail took as long as 20 minutes each, at that time we had analogue telephone modem connection. From the 3rd VHACP we bought a Cybershot Sony 1.3 Megapixel (mpx) digital camera with 3× optical zoom, and got the photos directly from the eye of the microscope. Later we took photos with a Cybershot Sony 5.1 mpx, but at only 1 mpx. We used Corel Photo paint program for editing, compression and resized to 640 × 480 pixels (VGA). 9th VHACP, one case, was captured in a photomicroscope of the National Cancer Center of Tokyo, Japan, during JICA training course. In the VSCs, we sent the slides by airmail, prior to approval of cases by the scientific committee. Since 7th VHACP we had broadband ADSL Internet connection and in the recent Congresses with the online autoeditor we "fill each entire work", enclosing pictures after JPG compression, the process becoming easier and faster.

## Results

In the VHACP, we presented a total of 18 publications in ST [3] and in the 1st and 2nd Internet VSCs two of our cases were digitized in Spain (case 1 and 3 respectively) [2]. In addition to the certificates as author and CDs or DVDs of the Congresses, in the last VHACP a self-evaluation test of 50 questions, or more than 50 hours of reading and 5 opinions in the active phase of the congress can be certified 8.6 credits, which is equivalent to 50 hours for continuing Medical Education, recognized by SEAFORMEC (English System of Continuous Medical Education) and by the European Accreditation Council for CME, an official body of the European Association of Medical Specialists (UEMS) [2].

## Discussion

The active participation in ST from anywhere in the world, allows easy exchange of knowledge. The digital photos are now available practically to any pathologist in the world, with common digital cameras, taking images directly from the ocular of the microscope, which is more understandable in developing countries by the lack of sufficient resources to obtain photomicroscopes with specialized software [3]. At this point it is important to note that there is no requirement to have the latest generation of cameras, nor with the highest mpx, since they only create greater size of image and therefore a larger file (weight of the photo), downloading by the Internet will be very slow. ADSL 640 Kbps connection can download a photo of 80 kbytes in a second, therefore when a pathologist is considering the first photo; the other images are downloaded to the computer monitor. The preparation of the sessions in recent VHACP is easier because the autoeditor intuitively guide the steps for editing [2]. The Virtual Congresses have the advantage of presenting virtually endless amounts of pictures free, with the added value of having the ability to have access to them any time of day or night, without taking into account time differences between countries, there is no need to travel, because "one has to go so far as to the place of your computer." In the VSCs, the slides can be moved remotely from any computer connected to the Internet, the use of a basic ADSL connection is easy as we use it with any image, just a little more time, almost imperceptible, is needed to view the images [3]. With these characteristics we should recognize that it will become a most valuable tool in continuing Medical Education by microscopy [2], probably related to the phrase "a picture is worth more than a thousand words", and we might add: then "what about thousands of photos", as a virtual slide, virtually identical to a real slide in a microscope? Similarly, the autoevaluation test and the participation in forums give an enriching experience.

## Conclusion

Static and Dynamic Telepathology, in the support of Virtual Congresses allows learning, teaching and sharing of diseases in scientific presentations, and the exchange of views in the forums. As a whole, they are the optimum material for Distance Education. Digital images of Pathology can be obtained virtually by any pathologist in the world who must feel encouraged to share (and teach) his/her interesting cases, for all of us to learn. The active participation and the autoevaluation test are the best tools for a Continuous Medical Education in Telepathology, not only for pathologists in developing countries, but also for the entire world.
